# Experimental Study on the Lateral Seepage Characteristics in the Tension Saturated Zone

**DOI:** 10.3390/ijerph18105098

**Published:** 2021-05-12

**Authors:** Yongfeng Gong, Zuo Liu, Chuanming Ma, Minghong Li, Xu Guo

**Affiliations:** 1 School of Environmental Studies, China University of Geosciences, Wuhan 430074, China; gyf221@126.com (Y.G.); liuzuo0206@cug.edu.cn (Z.L.); machuanming@cug.edu.cn (C.M.); liminghong98@cug.edu.cn (M.L.); 2 Ningxia Institute of Land and Resources and Monitoring, Yinchuan 750002, China

**Keywords:** lateral seepage, tension saturated zone, thickness of the tension saturated zone, physical experiment, water potential

## Abstract

To study the lateral seepage field in the tension saturated zone (TSZ), an experiment with no evaporation and precipitation infiltration was carried out in a self-made seepage tank filled up with fine sand. Based on the data and plots obtained, the lateral seepage field distribution features in the TSZ can be divided into three area for discussion: ascending area, descending area, and the nearly horizontal flow area. In the ascending and descending area, the total water potential gradient diminished from the recharge area to the discharge area and the seepage velocity was faster. In the nearly horizontal flow area, the total water potential gradient was lower and the seepage velocity was slower. The pressure potential gradually decreased horizontally from the recharge area to the discharge area, while in the vertical profile, it gradually decreased from the bottom to the top in the whole seepage area. In the absence of evaporation, the vertical water exchange among the saturated zone, TSZ, and unsaturated zone in nearly horizontal flow area is weak. Contrarily, in the ascending area and descending area, vertical water flows through both the phreatic surface and the upper interface of the TSZ. When there is lateral seepage in the TSZ, the thickness of the TSZ generally increases from the ascending area to the nearly horizontal area and then to the descending area. It should be pointed out that in the nearly horizontal area, the TSZ thickness is approximately equal to the height of the water column. Overall, the lateral seepage in the TSZ can be regarded as a stable siphon process, hence the siphon tube model can be further used to depict this lateral seepage.

## 1. Introduction

Lateral flow research in the unsaturated zone began in the middle of the last century [1]. However, lateral seepage in the tension saturated zone (TSZ) is often underestimated due to the fact that the TSZ is relatively thinner than the saturated zone [2]. In recent years, public concern has increased over the migration characteristics of pollutants in soil and groundwater [3]. The TSZ has been proved to be the crucial way for contaminants from the surface to enter into groundwater, and the lateral flow of the TSZ plays an important role in transporting contaminants in an aquifer system [4,5,6,7].

Field experiments involving pollutant tracers support the importance of lateral seepage of the TSZ; Freitas and Barker (2011) confirmed the fuel mixtures released into the unsaturated zone (at the CFB Borden aquifer) accumulated in the TSZ as they are lighter than water [8]. Lateral flow and transport in the TSZ of both ethanol and hydrocarbon compounds was significant, they emphasized that ethanol exclusively transported above the water table. In field experiments of Abit et al. (2008), the movement of both NO_3_^−^ and Br^−^ was monitored for 84 days by using tension lysimeters installed at depths at radial distances [9]. They found that plumes of nitrate and Br^−^ injected in the TSZ continued to migrate horizontally, until the water level fluctuations gradually brought both species partially into the saturated zone following rainfall events. Additionally, in their later field experiments, they highlighted that the surface-applied NO_3_^−^ transported laterally in the TSZ should be taken account [10].

Laboratory experiments on a seepage tank used to characterize the lateral flow of the TSZ with a phreatic surface slope are of great theoretical value as well. Silliman et al. (2002) observed lateral flow in the TSZ and described it in detail based on a sandbox experiment [3]. The results were obtained from image analysis at time series of tracer migration, the flow patterns in the TSZ can be simply described as follows: the main flow path is upward near the recharge area, downward near the discharge area, and nearly horizontal in the central area. Mazdeh and Wohnlich (2019) investigated the flow patterns in the TSZ by using a small-scale glass tank [11]. The flow velocity in the TSZ was quantitively delineated by tracking a fluorescent dye tracer at different depths of the TSZ. The results showed the flow velocity increased almost linearly toward the groundwater table. Moreover, they obtained conclusions similar to those of Silliman et al. (2002) and highlighted that lateral flow of the TSZ accounts for 35% of the total water flow, which presents that lateral flow of the TSZ plays a significant role in the transport of infiltrated solutes from the surface [3]. Ma et al. (2017) carried out a series of one-dimensional seepage experiments by using different types of porous media to discuss the seepage law of the TSZ [12]. When the moisture content ratio of the TSZ is equal to that of the saturated zone, they quantitatively discussed whether the TSZ and the saturated zone have the same seepage law and checked whether there existed a gradient of starting water potential. However, due to the difficulties in obtaining measurements in the TSZ [11], there are still some lacks in the quantitative characterization of water potential at present. The seepage law of the TSZ can be further discussed based on the scale of laboratory experiments.

In this study, a special conceptual model of interfluve was introduced to carry out the physical simulation experiment of TSZ [13]. The ceramic probing head of the tension meter was designed to measure the water potential in the flow field, which solves the measurement difficulty in TSZ. The aim of this study was to quantitatively describe the lateral seepage field distribution features in the TSZ (including water potential distribution features, distribution characteristics of the TSZ thickness, interfacial characteristics of the TSZ, etc.) under steady water potential boundary conditions.

## 2. Materials and Methods

### 2.1. Experimental Apparatus

**Figure 1:**
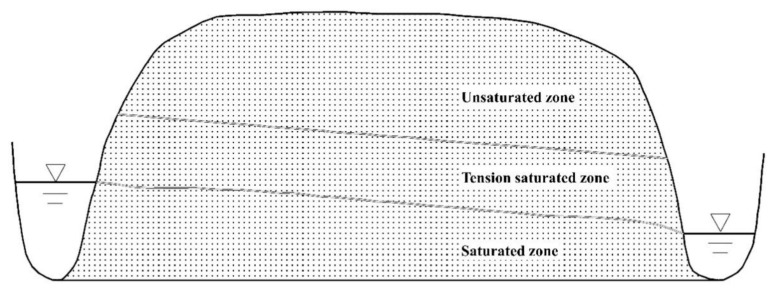
The conceptual interfluve model of the seepage zone.

In this study, the research of the lateral seepage field in the TSZ was based on the following conceptual interfluve model (Figure 1): 

(a) The interfluve, where the aquifer media was assumed to be homogeneous and isotropic, was defined as a cuboid seepage zone that lay between two parallel rivers [13].

(b) The two parallel rivers cut through the aquifer, and the bottom of the aquifer was flat.

(c) From bottom to top, the porous media consists of the saturated zone, TSZ, and unsaturated zone.

(d) Evaporation at the soil surface was ignored.

(e) The seepage was simplified as a 2D vertical profile flow.

**Figure 2:**
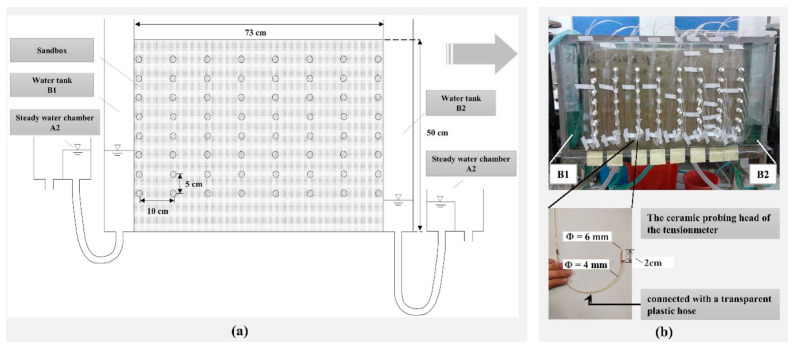
(**a**) The experimental apparatus for physical simulation; (**b**) object diagram of the experimental apparatus for physical simulation.

Based on the conceptual model (Figure 1), the experimental apparatus was made for the physical simulation:

(1) The experimental apparatus (73 cm long, 30 cm thick, and 50 cm tall) was an acrylic sandbox connected with two water tanks (B1, B2) (Figure 2a,b). The water level of B1, B2 could be controlled by two steady water chambers A1 and A2. Holes were horizontally distributed on the surface of the sandbox for placing probes. In this experiment, the total water potential was measured with the ceramic probing head of the tension meter inserted at different holes in the porous media (Figure 2b).

(2) We chose fine sand as the porous media for this experiment. The fine sand was uniformly and compactly loaded into the sandbox with a thickness of 50 cm, which can be regarded as homogeneous and isotropic. The operating parameters were as follows:

Dry density of fine sand = 1.49 g/cm^3^; air-entry value = 18.0 cm water column; saturated permeability coefficient under positive pressure = 7.0 × 10^−4^ cm/s; porosity = 0.439; specific yield = 0.414; percentage of saturated water content θs = 0.439; percentage of residual water content θr = 0.025.

**Figure 3:**
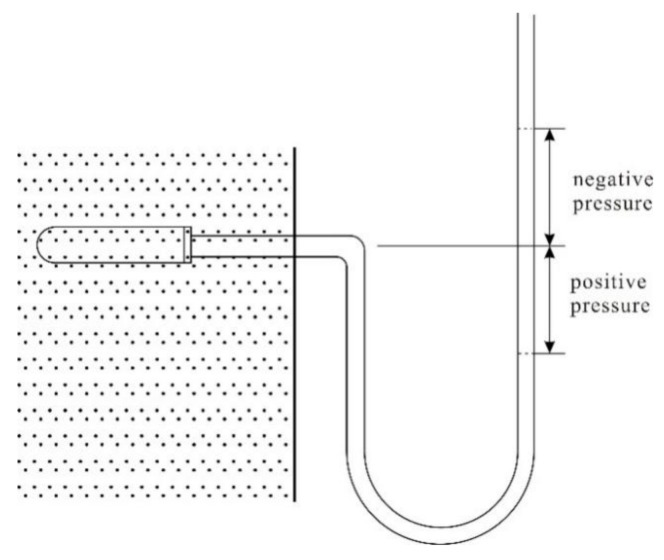
Schematic diagram of the tensiometer.

(3) The ceramic probing head of the tension meter used in the experiment was 6 mm in diameter and 2 cm long (Figure 2b). It was connected with a transparent plastic hose with a 4 mm diameter and 1 m long. Because the difference in the total water potential at various points in the seepage field of this experiment was small (0–7 cm), it was difficult to observe with a mercury manometer. Thus, the water column height was used to reflect the total water potential, so the transparent plastic hose was bent into a U-tube as shown in Figure 3.

When the pressures on the two sides of the U-tube were balanced, if the piezometric tube level was lower than the position of the ceramic probing head of the tension meter position, the potential near the ceramic probing head of the tension meter showed negative pressure (atmospheric pressure was taken as the benchmark). If the piezometric tube level was higher than the ceramic probing head of the tension meter position, the potential near the ceramic probing head of the tension meter showed positive pressure. The absolute value of pressure potential is the water column height corresponding to the elevation difference between the piezometric tube level and the position of the ceramic probing head.

The bottom elevation of the sandbox was taken as a gravitational potential benchmark, and the total water potential near the ceramic probing head of the tension meter is the elevation difference between the piezometer tube and the sand box bottom.

### 2.2. Experimental Conditions and Process

#### 2.2.1. Experimental Conditions

During the experiment, the surface of the sand layer was covered with plastic film to prevent evaporation. Ordinary tap water was used in the experiment. The laboratory temperature was maintained at 18–20 °C. A stainless steel ruler was used for measurement in the experiment.

#### 2.2.2. Experimental Process

The experimental process is as follows:

(1) Saturation of sand layer

(a) Adjust the movable water tank to form the same low water level on both sides of the seepage box.

(b) Raise the water level on both sides and slowly saturate the sand to drive out air until the sand is completely saturated.

(2) Commissioning of the pressure measuring tube

Use a syringe to slowly extract air from the tube until it is filled with water.

(3) Adjust the water level difference

This step is the key to the formation of the tension saturated zone.

(a) Gradually reduce the water level on one side, to create the water level difference on both sides of the flume.

(b) Reduce the water level on both sides until the water level on both sides reaches about 20 and 10 cm, respectively. Then keep the water level constant on both sides.

(c) Measure the elevation difference between the water level of the pressure tube of each tension gauge and the bottom plate of the seepage box every hour. When the value does not change for 10 consecutive hours, the whole system is considered to be stable.

(4) Data record

When stable, record the data of each pressure measuring tube. Under this condition, the elevation difference between the water level of the tension gauge pressure tube and the bottom plate of the seepage box is the total water potential at the corresponding position (Ψ).

### 2.3. Data Processing

**Figure 4:**
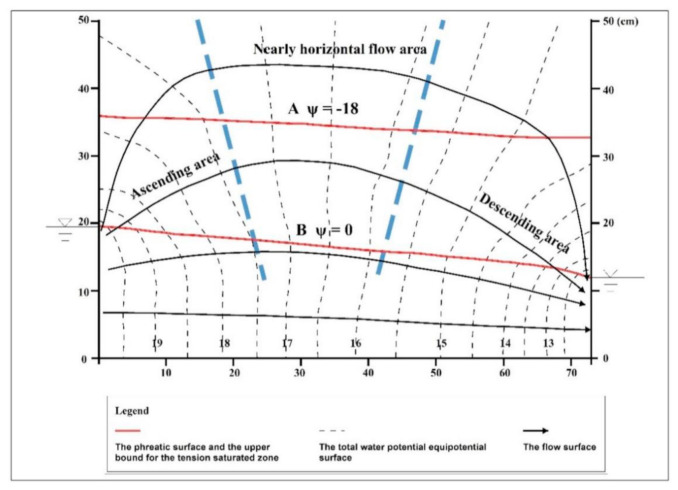
Flow net of the seepage zone as measured in the physical simulation.

**Figure 5:**
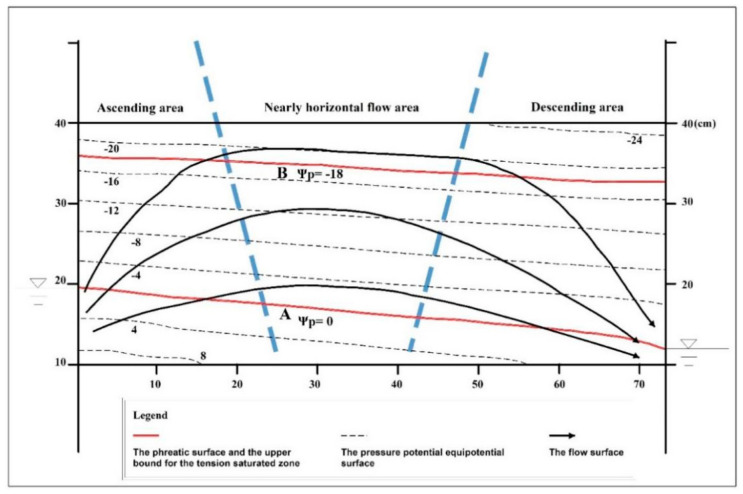
Pressure potential distribution as measured in the physical simulation.

The total water potential at each point and the corresponding position parameters were input into Surfer software for drawing, and the distribution map of the total water potential equipotential surface and pressure equipotential surface in the stable seepage field was obtained (Figure 4 and Figure 5). Then the flow surface was drawn according to the law that the flow surface is perpendicular to the equipotential surface, and the flow network diagram of the percolation zone was obtained. It showed that the distribution of the flow surface was consistent with the previous experimental results [3,11].

Among them, the surface with zero pressure potential (A) is the diving surface, and below it is the saturation zone. The equipotential surface (B) of the pressure potential corresponding to the intake value is the upper boundary of the capillary saturation zone, and the upper boundary is the unsaturated zone.

## 3. Results and Discussion

### 3.1. Water Flow Characteristics

The flow field in the TSZ could be divided into three parts (Figure 4):

(1) Around the recharge area, where the water flow was mainly upward. In this study, this area was called the “Ascending area”.

(2) Near the discharge area, where the water flow was mainly downward. In this study, this area was called the “Descending area”.

(3) In the middle of the flow field, where the water flow was nearly horizontal. This area was called the “Nearly horizontal flow area”.

### 3.2. Water Potential Distribution Features of the Seepage Field

#### 3.2.1. Total Water Potential Distribution Features

Under the steady flow of water in the porous media, as shown in Figure 4, the total water potential demonstrated the following characteristics.

(1) In the ascending area, the equipotential surface of the total water potential appeared as a series of curved surfaces that were normal to the recharge source. The total water potential decreased from the inside out, while the total water potential gradient decreased gradually from bottom to top. Near the recharge areas, the total water equipotential surface and stream surface showed a dense distribution. The water potential gradient was higher and the seepage velocity was faster in these areas.

(2) In the descending area, the equipotential surface of the total water potential appeared as a series of curved surfaces that were normal to the discharge area, and the total water potential decreased from the outside in. The total water potential gradient decreased gradually from bottom to top. Near the discharge areas, the total water equipotential surface and stream surface showed a dense distribution. The water potential gradient was higher and the seepage velocity was faster in these areas.

(3) In the central nearly horizontal flow area, the equipotential surfaces of the total water potential appeared as a series of approximate vertical and parallel planes along the horizontal direction. The total water potential weakly changed in the vertical direction and mostly changed in the horizontal direction. The total water potential diminished from the recharge area to the discharge area, the total water potential gradient was lower than that of the other two flow area, and the seepage velocity was slower.

#### 3.2.2. Pressure Potential Distribution Features

It is assumed that when the water did not flow in the porous media, the pressure potential gradient was in the vertical direction and equal to 1. Then, the pressure potential (expressed as the water column height) for a position at a vertical distance above the phreatic surface was equal to that for the position at the opposite vertical distance.

When the water flow was stable and lateral in the porous media, as shown in Figure 4, the pressure potential distribution was as shown in Figure 5. Table 1 and Table 2 compare the pressure potential distributions of the three parts based on the physical simulation results. Horizontally, the pressure potential tended to weakly decrease from one side of the recharge area to the other side of the discharge area. The pressure potential gradient barely changed.

As shown in Figure 5 and Table 1 and Table 2, in the central nearly horizontal flow area, the vertical pressure gradient was approximately equal to the pressure gradient when the water was resting (approximately equal to 1). The pressure potential (expressed as the height of water column) for a position at a vertical distance above the phreatic surface was approximately the same when the phreatic surface is stable. Its value is approximately the negative of the vertical distance. In the ascending area, the vertical pressure gradient was higher than that of the central nearly horizontal flow area and still water, and the pressure potential equipotential surface was denser. The pressure potential in this zone was lower than that in the central nearly horizontal flow area and in still water at the same vertical distance above the phreatic surface. In the descending area, the vertical pressure gradient was lower than that of the central nearly horizontal flow area and still water, and the pressure potential equipotential surface was sparser. The pressure potential in this area was higher than that in the central nearly horizontal flow area and in still water at the same vertical distance above the phreatic surface.

The distribution characteristics were analyzed as follows:

Take the vertical upward direction as the positive direction. Then, the relationship between the variations of the vertical pressure potential and total water potential is as follows:

(1) $$\frac{\partial \psi }{\partial z}=\frac{\partial \psi_{p}}{\partial z}+\frac{\partial \psi_{z}}{\partial z}=\frac{\partial \psi_{p}}{\partial z}+1$$

where  ψ, $\psi_{p},$ and $\psi_{z}$ are the total water potential, pressure potential, and gravity potential, respectively.

The pressure potential decreases from bottom to top, so the vertical gradient of the pressure potential is as follows:

(2) $$\left|\frac{\partial \psi_{p}}{\partial z}\right|\;=-\frac{\partial \psi_{p}}{\partial z}$$

The pressure potential at the vertical distance *z*_0_ above the phreatic surface is as follows:

(3) $$\psi_{p_{z0}}=0-z_{0}\cdot \left|\frac{\partial \psi_{p}}{\partial z}\right|\;=-z_{0}\cdot \left|\frac{\partial \psi_{p}}{\partial z}\right|$$

When the water is resting, $\left|\frac{\partial \psi_{p}}{\partial z}\right|$=1, $\psi_{p_{z0}}=-z_{0}$, then:

(1) In the central nearly horizontal flow area, the vertical total water potential is approximately constant, namely:

(4) $$\frac{\partial \psi_{p}}{\partial z}+1\approx 0$$

Therefore,

(5) $$\left|\frac{\partial \psi_{p}}{\partial z}\right|\;=-\frac{\partial \psi_{p}}{\partial z}\approx 1$$

The vertical pressure gradient is approximately equal to 1 and shows the same value as when the water is resting.

The pressure potential at the vertical distance *z*_0_ above the phreatic surface is as follows:

(6) $$\psi_{p_{z0}}=-z_{0}\cdot \left|\frac{\partial \psi_{p}}{\partial z}\right|\;\approx -z_{0}$$

Thus, the pressure potential at the vertical distance *z*_0_ above the phreatic surface is equal to that in the still water.

(2) For the ascending area, the vertical gradient of the total water potential is in the upward direction, and the absolute value is $\left|\frac{\partial \psi }{\partial z}\right|=-\frac{\partial \psi }{\partial z}$. According to Equations (1) and (2),

(7) $$\left|\frac{\partial \psi_{p}}{\partial z}\right|\;=-\frac{\partial \psi_{p}}{\partial z}\;=1+\left(-\frac{\partial \psi }{\partial z}\right)=1+\left|\frac{\partial \psi }{\partial z}\right|\;>1$$

The pressure potential vertical gradient is equal to 1 plus the absolute value of the water potential gradient in the vertical direction. This is greater than that of the central nearly horizontal flow area and still water.

The pressure potential at the vertical distance *z*_0_ above the phreatic surface is as follows:

(8) $$\psi_{p_{z0}}=-z_{0}\;\cdot \;\left|\frac{\partial \psi_{p}}{\partial z}\right|\;<-z_{0}$$

This indicates that the pressure potential at the vertical distance *z*_0_ above the phreatic surface is less than that of a same position above the phreatic surface in the central nearly horizontal flow area and in still water.

(3) For the descending area, the vertical gradient of the total water potential is downward, and the absolute value is $\left|\frac{\partial \psi }{\partial z}\right|=-\frac{\partial \psi }{\partial z}$. According to Equations (1) and (2),

(9) $$\left|\frac{\partial \psi_{p}}{\partial z}\right|\;=-\frac{\partial \psi_{p}}{\partial z}=1-\frac{\partial \psi }{\partial z}=1-\left|\frac{\partial \psi }{\partial z}\right|\;<1$$

The vertical gradient of the pressure potential is equal to 1 minus the absolute value of the water potential gradient in the vertical direction. This is less than that of the central nearly horizontal flow area and still water.

The pressure potential at the vertical distance *z*_0_ above the phreatic surface is as follows:

(10) $$\psi_{p_{z0}}=-z_{0}\cdot \left|\frac{\partial \psi_{p}}{\partial z}\right|\;>-z_{0}$$

In other words, the pressure potential at the vertical distance *z*_0_ above the phreatic surface is greater than that of a same position above the phreatic surface in the central nearly horizontal flow area and in still water.

### 3.3. Distribution Characteristics of the TSZ Thickness

The TSZ thickness refers to the vertical distance from the phreatic surface to the upper bound of the TSZ. The TSZ thickness for each area can be as given in Table 3.

The upper bound of the TSZ is the pressure potential equipotential surface that is equal to the air-entry value. The phreatic surface is in fact the equipotential surface pressure potential with zero potential. Then, the TSZ thickness *d_scf_* is given by

(11) $$d_{scf}=\frac{0-p_{d}}{\bar{\left|\frac{\partial \psi_{p}}{\partial z}\right|}}$$

where $p_{d}$ is the porous medium’s air-entry value and $\bar{\left|\frac{\partial \psi_{p}}{\partial z}\right|}$ is the average vertical pressure potential gradient.

When the water in the porous media is still, because the vertical pressure potential gradient is equal to 1, $d_{scf}=\;\left|p_{d}\right|$. When there is seepage as shown in Figure 4, in the central nearly horizontal flow area, $\bar{\left|\frac{\partial \psi_{p}}{\partial z}\right|}\approx 1$ according to Equation (5), then according to Equation (11), $d_{scf}\approx \;\left|p_{d}\right|$. In the ascending area, $\bar{\left|\frac{\partial \psi_{p}}{\partial z}\right|}>1$ according to Equation (7). then according to Equation (11), $d_{scf}<\;\left|p_{d}\right|$. In the descending area, $\bar{\left|\frac{\partial \psi_{p}}{\partial z}\right|}<1$ according to Equation (9), then according to Equation (11), $d_{scf}>\left|p_{d}\right|$. According to the physical simulation results (air-entry value is the height of a water column of −18 cm), according to Equation (1), the TSZ thickness for each area can be obtained (Table 3).

In summary, when the groundwater in the porous media is in a stable state, the TSZ thickness is equal to the air-entry value of the water column height. The thickness of TZT in the middle nearly horizontal flow area is approximately equal to the air-entry height of the water column when there is transverse seepage in the TZT. The TSZ thickness of the ascending area is less than the air-entry value of the water column height and greater than that in descending area. The TSZ gradually thickens from the ascending area to descending area.

### 3.4. Interfacial Characteristics of the TSZ

#### 3.4.1. Characteristics of the Phreatic Surface and Upper Bound of the TSZ

As shown in Figure 5, in the near horizontal flow area, both the phreatic surface and the upper interface of the TZT can be approximately regarded as the flow surface. In addition, under the condition of no evaporation, the vertical water exchange among saturated area, the TZT, and the unsaturated area is poor.

For both the ascending area and the descending area, vertical water seepage exists in the phreatic surface and the upper bound of the TSZ, so they are not flow surfaces. In the ascending area, the water goes from the saturation area through the phreatic surface into the TSZ. Some of the water is oriented into nearly horizontal lateral seepage in the TSZ, while the other part goes upward through the upper bound of the TSZ into the vadose area. In the descending area, there is a constant water flow through the upper bound of the TSZ down into the TSZ. It comes together with the water of the nearly horizontal lateral seepage area in the TSZ and then flows through the phreatic surface down into the saturation area.

#### 3.4.2. Characteristics of the Lateral Boundary of the TSZ

**Figure 6:**
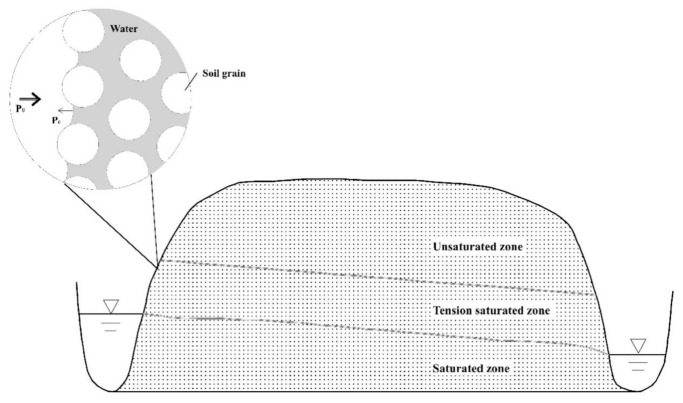
Pore meniscus in the water–vapor interface of the seepage area (modified by [14]).

A wide pore forms a concave surface on the upper bound of the TSZ (Figure 6). The air-entry value is inversely proportional to curvature of the pore. A narrow pore at this position does not form a concave surface and is still full of water, because the height of the capillary rise is greater, as well as because the air–water interface is at a higher position.

Figure 6 shows the water distribution at the air–water interface at the lateral boundary of the TSZ and near the recharge and discharge areas. Because of the negative pressure potential of the TSZ (the atmospheric pressure was taken as the benchmark), the lateral boundary of the air–water surface is concave toward the air side, and the bending degree is negatively correlated to the nearby pressure potential. Because of the decrease in the pressure potential from bottom to top, the concave curvature of the same pore aperture on the side wall surface increases from bottom to top. Because the air–water surface at the side wall is normal to the concave surface of the gas phase, it can be a flow surface, and water cannot flow in and out through the TSZ side walls.

#### 3.4.3. Causes of the Lateral Seepage Characteristics

The above experimental results show that when there is a difference in the water level of the free surface on both sides of the TSZ, lateral seepage is present in the TSZ. As shown in Figure 4 and Figure 5, lateral seepage is a process in which the water flows along the inverted U-shaped channels from the high water level to the low water level in the saturated area of negative pressure, that is, the lateral seepage in the TSZ is essentially a siphon process [15].

Usually, the siphon requires a closed tube wall, and the water is not connected to the atmosphere. Because the pressure is in a negative state in the siphon, once the tube wall is perforated, due to the different pressure inside and outside the siphon, gas will continue to fill the siphon until the siphon process is interrupted. However, the porous media’s porosity channels are connected to the atmosphere. The reason for the siphon process in the TSZ being sustained may be that the concave surface of the pore water resists the different pressure between the inside and the outside. A capillary bundle siphon experiment was conducted to verify the theory.

**Figure 7:**
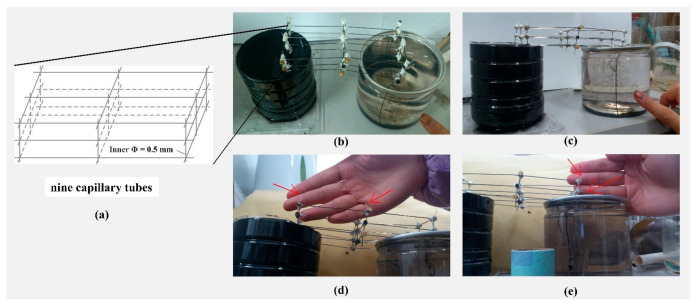
(**a**) Schematic diagram of nine capillary tubes; (**b**–**e**) the capillary bundle siphon experiment.

As shown in Figure 7a, nine capillary tubes with an inner diameter of 0.5 mm and a maximum water rising height of 5.5 cm were arranged in parallel to form a capillary bundle. The end of every capillary tube was open to the atmosphere. After the capillary bundle was saturated, the lower end of the capillary tubes on both sides were inserted into different water tanks while maintaining the vertical height of the tube bundle not exceeding the maximum rising height of the capillary tube. Then, a certain amount of black ink was injected in the higher water tank as a tracer (Figure 7b,c).

The experimental phenomena and analysis are as follows:

(1) The stained water in the high water tank continuously flows through the capillary bundle into the low water tank, indicating the presence of siphon flow in the capillary bundle.

(2) The siphon process of the capillary bundle is continuously stable, and air cannot enter the siphon capillary bundle from the capillary end opening to the atmosphere, indicating that the concave surface produces an additional pressure to effectively resist the internal and external pressure difference.

(3) There is no tracer flow in the vicinity of the capillary end where the concave surface is located (see the position indicated by the red arrow in Figure 7d,e), indicating that there is no flow of water there, where there is no water potential gradient. Therefore, the capillary action is not the cause of the capillary bundle siphon flow, but it only provides a pressure condition for the continuous siphon process.

Therefore, the lateral seepage in the TSZ can be seen as a capillary bundle siphon process. During the lateral seepage in the TSZ, the concave surface resists the internal and external pressure difference, that is, the capillary effect provides a guarantee of pressure conditions for the maintenance of the siphon process. However, the capillary effect is not the power source of the lateral seepage in the TSZ. The fundamental cause of this seepage is the water potential difference on both sides of the siphon.

## 4. Conclusions

Based on the observation of the lateral seepage characteristics in the TSZ, the following water flow characteristics were determined:

(1) The total water potential characteristics are described as follows: (a) In the ascending area, the total water potential and the total water potential gradient decrease gradually from the inside out and from the bottom up, respectively. The water flow from the recharge area is mainly upward, with faster seepage velocity. (b) In the descending area, the total water potential and the total water potential gradient decrease gradually from the outside in and from the bottom up, respectively. The water flow to the discharge area is mainly downward, with faster seepage velocity. (c) In the nearly horizontal area, the total water potential decreases from the recharge area to the discharge area, and the water flow is nearly horizontal, with slower seepage velocity.

(2) Horizontally, the pressure potential decreases slightly from the recharge area to the discharge area. The vertical profile of the pressure potential gradually decreases from the bottom to the top for the whole seepage area.

(3) When there is a lateral seepage in the TSZ, the thickness of the TSZ generally increases from the ascending area to the central nearly horizontal flow area to the descending area. In the central nearly horizontal flow area, the TSZ thickness is approximately equal to the water column height.

(4) The upper boundary of the TSZ can be approximated as a flow surface in the nearly horizontal flow area. The lateral boundaries of the TSZ can be also defined as flow surfaces.

(5) Lateral seepage is a process in which the water in the saturated negative pressure area flows along the inverted U-shaped channels from the high water level to the low water level. The siphon flow experiment of the capillary glass tube shows that the concave surface of the capillary can resist the internal and external pressure difference and ensure the continuation of the siphon process. The water potential difference on both sides of the capillary area provides power to the lateral seepage.

The lateral seepage characteristics in the TSZ have shown there is a unified seepage field made up of the TSZ and the area of saturation and supported the fact that capillary water can transfer both vertical and horizontal hydrostatic pressure. The lateral seepage in the TSZ is a stable siphon process. The siphon tube model can be used to depict this lateral seepage.

## Figures and Tables

**Table 1:** Comparison of vertical pressure potential gradients for each area.

Pressure Potential (Water Column: cm)	Ascending Area (e.g., Abscissa of 5 cm in x Axis)	Nearly Horizontal Flow Area (e.g., Abscissa of 30 cm in x Axis)	Descending Area (e.g., Abscissa of 65 cm in x Axis)
0 to −8 (the upper part of TSZ)	1.10	1.01	0.92
−8 to −18 (the lower part of TSZ)	1.07	0.99	0.97

**Table 2:** Comparison of pressure potentials for each area (cm).

Vertical Distance above Water Table	Ascending Area (e.g., Abscissa of 5 cm in x Axis)	Central Nearly Horizontal Flow Area (e.g., Abscissa of 30 cm in x Axis)	Descending Area (e.g., Abscissa of 65 cm in x Axis)
5	−5.68	−5.10	−4.57
10	−11.04	−10.15	−9.28
15	−16.47	−15.18	−14.08

**Table 3:** TSZ thickness for each area.

Area	Ascending Area (e.g., Abscissa of 5 cm in x Axis)	Central Nearly Horizontal Flow Area (e.g., Abscissa of 30 cm in x Axis)	Descending Area (e.g., Abscissa of 65 cm in x Axis)
Measured value	16.4	17.9	19.0
Calculated value	16.5	17.8	19.0

## Data Availability

No new data were created or analyzed in this study. Data sharing is not applicable to this article.
